# Supplementary material for: Causal Relationship between Aging and Anorexia Nervosa: A White-Matter-Microstructure-Mediated Mendelian Randomization Analysis
Source: Biomedicines. 2024 Aug 16;12(8):1874. doi: 10.3390/biomedicines12081874 (PMC11351342; doi:10.3390/biomedicines12081874)
Supplement: Supplementary file 1 [file biomedicines-12-01874-s001.zip › Supplementary Figure/Figure S4/Singe SNP plot of MR analysis for TL to ubm-b-1686.pdf]

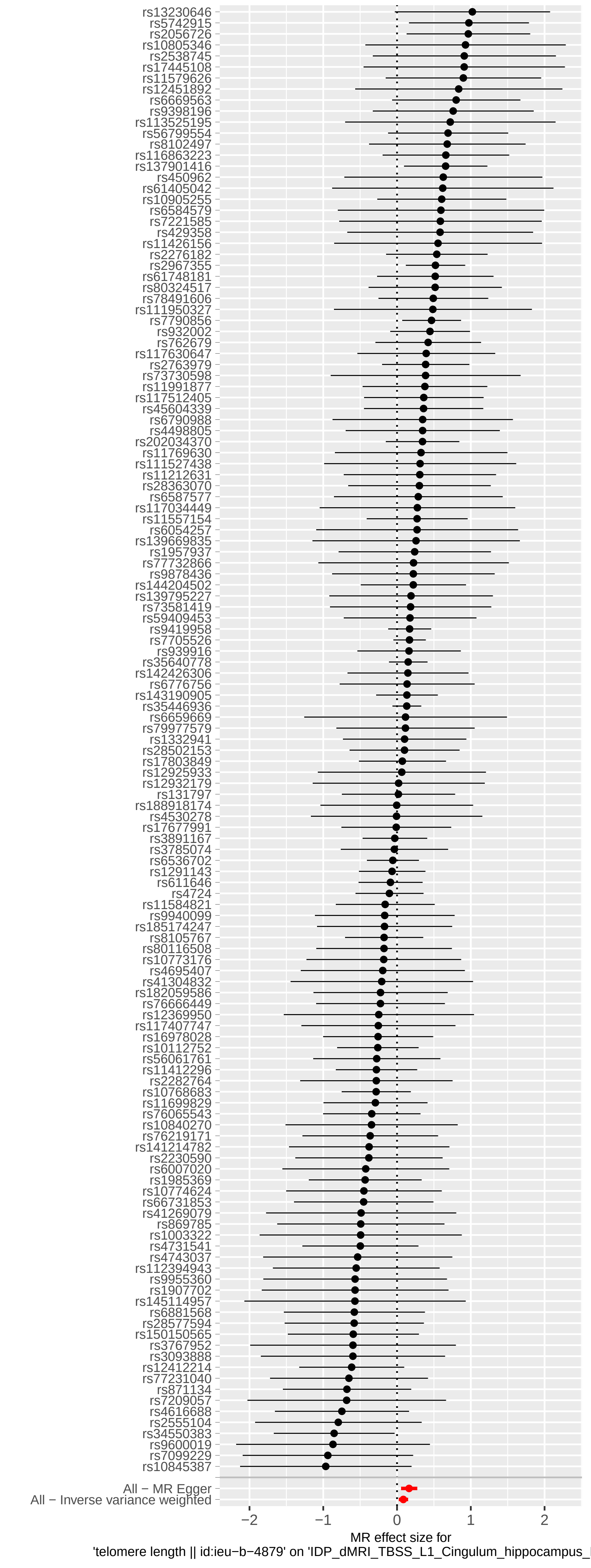
rs13230646
rs5742915
rs2056726
rs10805346
rs2538745
rs17445108
rs11579626
rs12451892
rs6669563
rs9398196
rs113525195
rs56799554
rs8102497
rs116863223
rs137901416
rs450962
rs61405042
rs10905255
rs6584579
rs7221585
rs429358
rs11426156
rs2276182
rs2967355
rs61748181
rs80324517
rs78491606
rs111950327
rs7790856
rs932002
rs762679
rs117630647
rs2763979
rs73730598
rs11991877
rs117512405
rs45604339
rs6790988
rs4498805
rs202034370
rs11769630
rs111527438
rs11212631
rs28363070
rs6587577
rs117034449
rs11557154
rs6054257
rs139669835
rs1957937
rs77732866
rs9878436
rs144204502
rs139795227
rs73581419
rs59409453
rs9419958
rs7705526
rs939916
rs35640778
rs142426306
rs6776756
rs143190905
rs35446936
rs6659669
rs79977579
rs1332941
rs28502153
rs17803849
rs12925933
rs12932179
rs131797
rs188918174
rs4530278
rs17677991
rs3891167
rs3785074
rs6536702
rs1291143
rs611646
rs4724
rs11584821
rs9940099
rs185174247
rs8105767
rs80116508
rs10773176
rs4695407
rs41304832
rs182059586
rs76666449
rs12369950
rs117407747
rs16978028
rs10112752
rs56061761
rs11412296
rs2282764
rs10768683
rs11699829
rs76065543
rs10840270
rs76219171
rs141214782
rs2230590
rs6007020
rs1985369
rs10774624
rs66731853
rs41269079
rs869785
rs1003322
rs4731541
rs4743037
rs112394943
rs9955360
rs1907702
rs145114957
rs6881568
rs28577594
rs150150565
rs3767952
rs3093888
rs12412214
rs77231040
rs871134
rs7209057
rs4616688
rs2555104
rs34550383
rs9600019
rs7099229
rs10845387
All − MR Egger
All − Inverse variance weighted
−2
−1
0
1
2
MR effect size for
'telomere length || id:ieu−b−4879' on 'IDP_dMRI_TBSS_L1_Cingulum_hippocampus_
